# From who we are to what we are willing to do for social change: The action‐bound role of efficacy perceptions

**DOI:** 10.1111/bjso.12910

**Published:** 2025-06-16

**Authors:** Alice Lucarini, Veronica Margherita Cocco, Loris Vezzali, Terri Mannarini, Huseyin Çakal

**Affiliations:** ^1^ Faculty of Medicine University of Modena and Reggio Emilia Reggio Emilia Italy; ^2^ Department of Human and Social Sciences University of Salento Lecce Italy; ^3^ School of Psychology Keele University Keele UK

**Keywords:** efficacy perceptions, normative and non‐normative collective action, social identification

## Abstract

Two cross‐sectional studies conducted in Chile (Study 1, *N* = 587) and Italy (Study 2, *N* = 438) investigated the action‐bound role of perceived efficacy in explaining the association between politicized (Studies 1 and 2) and non‐politicized identity (Study 2) with normative and non‐normative collective action (CA) intentions. We comparatively explored different efficacy perceptions: internal locus of control (i.e., individual agency), group efficacy (i.e., ingroup agency), normative and non‐normative collective action efficacy (i.e., action efficacy). Both identity types were positively associated with normative CA intentions via increased perceptions of group and normative CA efficacy (Studies 1–2). Regarding non‐normative CA intentions, beyond observing positive associations with politicized identity via increased group (Study 1) and non‐normative CA efficacy (Study 1–2), we also found a negative indirect effect of politicized identity via increased normative CA efficacy (Study 1) and a negative indirect effect of non‐politicized identity via decreased non‐normative CA efficacy (Study 2). These findings highlight the key role of efficacy perceptions in translating identity into action, emphasizing both group agency and the perceived efficacy of specific forms of action. Moreover, they suggest that the type of social identity can promote or inhibit more radical forms of CA, shaping pathways to social change.

## INTRODUCTION

In a world marked by both incidental and structural disadvantages that perpetuate oppression, understanding the driving forces that motivate people to undertake progressive collective action—collective efforts aimed at promoting and implementing social change—is of paramount importance. In this regard, one of the most powerful triggering factors of collective action emphasized by scholars in the last decades is social identity, defined as “that part of an individual's self‐concept which derives from his [or her] knowledge of his [or her] membership of a social group (or groups) together with the value and emotional significance attached to that membership” (Tajfel, 1978, p. 63; van Zomeren et al., 2008, p. 513). Serving as a catalyst for social change, social identity motivates people to undertake collective efforts. Several collective action models emphasized the prominent role of social identification in promoting mobilization (Becker & Tausch, 2015; Louis et al., 2020; Subašić et al., 2008; Thomas et al., 2009, 2012; van Zomeren et al., 2008, 2012, 2018). Among these, the social identity model of collective action (SIMCA; van Zomeren et al., 2008) and its integrations (van Zomeren et al., 2012, 2018), are widely recognized by scholars as a foundational framework for understanding collective action processes. SIMCA posits that social identification, particularly concerning politicized identities (activist identities linking group members to their disadvantaged status), can predict collective action, both directly and indirectly via increased perceptions of efficacy and injustice. Perceived injustice refers to group‐based experiences of inequality and can be distinguished into two main components: non‐affective injustice, which reflects the cognitive recognition of unfair treatment, and affective injustice, which involves the emotional response to injustice. Within this framework, affective injustice plays a more prominent role than non‐affective injustice, as emotions such as group‐based anger have been shown to be a stronger motivator of collective action (van Zomeren et al., 2008). In parallel, efficacy, particularly group efficacy, is crucial in driving collective action, as it refers to the collective belief that the group can transform its situation and destiny. Both affective injustice and group efficacy serve as key mechanisms that mobilize individuals to engage in collective efforts to address grievances (Mummendey et al., 1999; van Zomeren et al., 2008). However, the conceptualization and operationalization of perceived efficacy in collective action literature are unclear and varies within and across different research backgrounds (Hamann et al., 2024) resulting in a fragmentary comprehension of the construct. This complexity calls for a deeper understanding of the construct's various facets and impact on collective action (Uysal et al., 2024). Moreover, there is a lack of empirical evidence comparatively testing the predictive role of various operationalizations of the construct, precluding a proper understanding of their relative contribution to collective action (for exceptions see Saab et al., 2016; van Zomeren et al., 2013). Most importantly, there is no research examining comparative contribution of the construct's different operationalizations in explaining the association between distinct social identities and both normative and non‐normative collective action.

In light of these shortcomings, we conducted two cross‐sectional studies in culturally diverse settings—Chile and Italy—to examine the unique mediating contribution of three conceptually distinct forms of efficacy perceptions: internal locus of control (i.e., perceived individual agency), group efficacy (i.e., perceived ingroup agency), and normative and non‐normative collective action efficacy (i.e., perceived action efficacy). We considered both politicized (Studies 1 and 2) and non‐politicized identities (Study 2) as concurrent predictors to determine whether identification with different social identities operates through the same or different underlying processes in motivating collective action. In doing so, we also differentiated between normative (i.e., actions that align with prevailing social norms within a given context) and non‐normative collective action intentions (i.e., actions that deviate from these norms and can lead to more radical forms of action, which may or may not involve violence; Becker & Tausch, 2015). To provide a strict test for our hypotheses, we statistically accounted for the impact of affective injustice—a crucial mediator alongside efficacy posited by SIMCA—by including a measure of group‐based anger (van Zomeren et al., 2008). Results provide valuable theoretical insights into how social identification and perceived efficacy perceptions underpin and drive different forms of collective action, while also offering applied considerations concerning the potential instrumental use of such constructs to promote or sedate collective action.

### Social identification and collective action

Social identification has received long‐standing attention from scholars interested in understanding group processes, standing out as a pivotal element of intergroup behaviour (Hogg, 2016). This significance extends to its role in collective action literature, as underscored by prominent models in the field, which recognize it as a key driver of social change while delving into different approaches. SIMCA (van Zomeren et al., 2008, 2012, 2018) emphasizes the role of social identification, bringing particular attention to the powerful role of politicized identities in predicting collective action, both directly and indirectly, via perceived efficacy and injustice. Other popular models similarly highlight the role of social identification in predicting collective action. Among these, the encapsulation model of social identity in collective action (EMSICA; Thomas et al., 2009, 2012), while focusing on the same variables proposed by SIMCA, draws attention to the dynamic nature of collective action, postulating that the reverse path between social identification, perceived efficacy, and injustice is also plausible. Subašić et al. (2008), in their political solidarity model of social change, emphasize the significance of identification with superordinate groups (groups standing at a higher level of abstraction with respect to subgroups' identities, Gaertner & Dovidio, 2000), which may serve as a way out for the minority to ally with the majority group and challenge the status quo.

In any given social context, the social identity that motivates collective action may vary due to several factors, with identity orientation—the underlying goals and objectives associated with a social identity—being particularly crucial (van Zomeren et al., 2008). Identity orientation reflects the nature of the identity itself, as it dictates the goals that the group strives to achieve. For example, a politicized identity is characterized by a strong emphasis on political goals, where the primary aim is to challenge existing power structures, demand structural change or address grievances (Simon & Klandermans, 2001). Conversely, a non‐politicized identity may involve identifying with a disadvantaged group (van Zomeren et al., 2008), primarily focusing on protecting and preserving the group itself rather than engaging in political action. While non‐politicized identities may exist independently and mobilize when necessary, politicized identities emerge from the explicit intent to address specific grievances and are strongly goal‐oriented (Simon & Klandermans, 2001). In this regard, the nature of a social identity, inherently interconnected with its orientation and the specific goals it prioritizes, may shape the disposition of group members towards collective action—for example, determining whether it takes a normative or non‐normative form.

The positive association between social identity and normative collective action is consistently supported by numerous empirical studies (Cakal et al., 2011, 2016b, van Zomeren et al., 2008, 2012, 2018). However, in the case of non‐normative collective action, the scenario becomes more complex. There is evidence suggesting a negative relationship between social identity and more radical forms of collective action. Jiménez‐Moya et al. (2015), for instance, found that individuals with low levels of identification with Andalusians (a non‐politicized identity) were more inclined to support radical actions than those with high identification levels. The authors argued that this negative relationship may stem from the concerns of strongly identified individuals regarding the potential adverse consequences of less socially acceptable forms of collective action. Consequently, highly identified individuals may abstain from endorsing radical actions to avoid further compromising the group (e.g., reputational damage, Teixeira et al., 2020). Consistently, Stathi et al. (2019) found that national identification was associated with higher normative collective action intentions and lower support for non‐normative collective action intentions. In line with Jiménez‐Moya et al. (2015), the authors highlighted the potential adverse consequences of participating in more extreme actions that could have determined these results. However, they also attributed these findings to the specific form of identity involved, suggesting that national identification (i.e., a non‐politicized identity) may lead group members to strategically inhibit the intention to engage in non‐normative collective actions as a form of protection for the ingroup, driving individuals towards more constructive and socially acceptable actions. In other words, politicized and non‐politicized identities may shape the disposition of individuals towards non‐normative collective action, given their distinct goal orientation.

We therefore posit that the orientation of the identity involved —politicized or non‐politicized— may promote or prevent participation in non‐normative collective action. Politicized identities, originating and evolving based on specific goals within the power struggle, may drive group members to take extreme measures to achieve those goals (Obaidi et al., 2018; Wohl et al., 2014). In contrast, for individuals relying on non‐politicized identities, the identity focuses on the group rather than its goals may lead to greater concerns about the group image.

To disentangle their effects, it is important to conduct research testing the role of both politicized and non‐politicized identities in predicting collective action. By acknowledging their potentially different implications, here we include both politicized (Studies 1 and 2) and non‐politicized identity (Study 2).

### The key action‐bound role of perceived efficacy

Self‐efficacy, defined as the assessment of one's ability to carry out actions required to handle potential situations, lies at the core of human agency (Bandura, 1982). According to self‐efficacy theory, people's motivations are largely shaped by their beliefs rather than by objective circumstances (Bandura, 1995). The degree to which individuals perceive themselves as effective is closely tied to their prospective actions, influencing not only the likelihood of their engagement but also their effort and persistence in attaining short‐term and long‐term goals (Bandura, 1982). The action‐bound nature of efficacy perceptions (Hamann et al., 2024) extends to collective action, serving as a bridge between social identification and collective action (van Zomeren et al., 2008, 2012, 2018). Social identification qualifies as a significant predictor of perceived efficacy (van Zomeren, 2008, 2012, 2018), because strong identification can imbue individuals with a greater sense of agency, making them feel empowered agents of social change (Drury & Reicher, 2005), and thereby bolstering their intentions to engage in collective action (Mummendey et al., 1999). In other words, the likelihood of individuals engaging in collective action is directly proportional to the extent to which they perceive themselves, their group, and their actions as effective in achieving their goals (Hornsey et al., 2006, 2003; van Zomeren, 2008, 2012, 2018).

In the context of collective action research, specific forms of perceived efficacy have emerged as particularly relevant, and we review them in the next subsections.

#### Group efficacy

Many of the challenges faced by individuals concern group‐related issues that demand collective efforts to achieve social change. This underscores the significance of perceived group efficacy in the context of collective action, which consists of individuals' confidence in the ingroup's ability to achieve group goals through collective efforts (Bandura, 1982). Such a perception has the power to influence group decisions, actions, and resilience in the face of adversity, emerging as a pivotal form of efficacy able to motivate people to undertake collective action (Corcoran et al., 2011; Hornsey et al., 2006). While in the case of normative collective action results are consistent, showing a positive association between group efficacy and normative collective action (Çakal et al., 2016a; van Zomeren, 2008, 2012, 2018), findings are mixed for non‐normative collective action. On the one hand, there is evidence supporting the positive link between group efficacy and non‐normative collective action (Medel et al., 2022; Thomas et al., 2019). On the other hand, there are reasons to believe that an increased sense of group efficacy may not always translate into intentions to engage in non‐normative collective action, as individuals may engage in non‐normative forms only when normative strategies are considered ineffective (Louis, 2009; Pruitt & Gahagan, 1974). There is indeed evidence showing that non‐normative collective action can be triggered by a sense of low, rather than high, efficacy (Tausch et al., 2011). Tausch et al. (2011) argued that individuals may turn to non‐normative collective action when they perceive their situation as unlikely to improve, leading them to believe they have little or nothing to lose by reacting disruptively (see also Scheepers et al., 2006). Arguably, however, the negative relationship between perceived efficacy and non‐normative collective action may be more likely to emerge when considering individual‐, rather than group‐based, forms of perceived efficacy. This could be the case of internal locus of control, because of its direct correspondence with the individuals' perceptions of agency and autonomy in shaping their actions and life outcomes.

#### Internal locus of control

Internal locus of control — the extent to which individuals perceive themselves as able to exert influence over their own lives and destiny (Rotter, 1966) — represents a further construct that could promote individuals' engagement in collective action (Fukuzawa & Inamasu, 2020; Levenson & Miller, 1976). Empirical evidence shows that people with a strong internal locus of control are more inclined to actively address and resolve problems (Parkes, 1984). It was also found that those who engage in protests against social injustice generally show greater confidence in their ability to influence events in their lives than those who do not participate (Bandura, 1982). Consequently, an increase in internal locus of control can lead to greater motivation to address one's disadvantaged condition. In other words, perceiving control over one's actions, which can stem from belonging to empowering social identities, may foster the intention to engage in collective action to improve their circumstances.

However, as anticipated in the previous paragraph, in the case of non‐normative collective action, it is also possible that an increased sense of control may be associated with lower non‐normative collective action intentions, as more violent forms of actions tend to occur when disadvantaged groups feel powerless, lacking any sense of control over the situation (Tausch et al., 2011). Directly tackling individuals' perceptions of agency and autonomy, a high perception of internal locus of control may thus serve as a buffer, preventing individuals from resorting to more disruptive actions. Conversely, a lack of internal locus of control may be associated with greater intentions to undertake non‐normative forms of collective action, as individuals may perceive having nothing to lose (Scheepers et al., 2006). For instance, in the context of the anti‐racist Watts Riots that took place in the United States, Ransford (1968) demonstrated that feelings of powerlessness and a lack of control over events were positively linked to a willingness to engage in violence.

#### Collective action efficacy

The intention to engage in collective action may depend not only on the perceptions individuals have about their own and their group's capability to achieve social change but also on the perceived efficacy of the action itself. Such distinction becomes particularly relevant when considering different forms of collective action that people may undertake, extending beyond normative actions to encompass more violent actions (Saab et al., 2016). Following the expectancy‐value theory of behaviour (Fishbein & Ajzen, 1975), which posits that the belief that a certain behaviour will produce a desirable result increases the likelihood of engaging in that behaviour, the different forms of collective action may be predicted by their respective forms of action efficacy. Specifically, normative collective action efficacy may predict normative collective action intentions and non‐normative collective action efficacy could predict non‐normative collective action intentions (Hornsey et al., 2006). However, perceiving non‐normative collective action as effective may not automatically translate into intentions to engage in such actions, as individuals might resort to non‐normative forms only when normative actions are considered ineffective (Louis, 2009; Pruitt & Gahagan, 1974).

Conversely, but complementary, previous research suggests that, under certain conditions, the perceived efficacy of peaceful actions can predict lower support for aggressive forms of collective action (see Saab et al., 2016). Arguably, the choice to undertake aggressive versus peaceful actions in light of the perception of efficacy towards these actions is not mutually exclusive: individuals might consider aggressive and peaceful actions as complementary strategies (Uysal et al., 2024; Zúñiga et al., 2023), adopting them together to increase the likelihood of achieving social change. This scenario underscores the nuanced role of perceived action efficacy in predicting collective action, highlighting the importance of considering not just the agent of collective action but also how the action is perceived in terms of its (un)likely success.

### The present research

To extend previous literature, we aim to conduct a comparative test of the predictive role of both politicized (Study 1 and 2) and non‐politicized (Study 2) identities on normative and non‐normative collective action, while considering distinct forms of efficacy perceptions (group efficacy, internal locus of control, and normative and non‐normative action efficacy) as parallel mediators. To increase the external validity of our findings, we conducted our studies in two culturally distinct social contexts: a WEIRD (Western, Educated, Industrialized, Rich, and Democratic) society (Italy) and a non‐WEIRD context (Chile). Additionally, to avoid the risk of inflating results, we statistically controlled for group‐based anger, a strong predictor of collective action (van Zomeren et al., 2008).

### Hypotheses

#### Social identification and collective action intentions

We anticipate a direct positive association between politicized identity (Studies 1 and 2) and both normative and non‐normative collective action intentions.

For non‐politicized identity (Study 2), we anticipate a positive association with normative collective action intentions and a negative association with non‐normative collective action intentions (to protect the ingroup from potential damage due to involvement in destructive actions).

#### Indirect effects

Concerning normative collective action, politicized identity (Studies 1 and 2) is expected to show positive indirect associations via increased perceived efficacy perceptions, except for non‐normative collective action efficacy. Instead, non‐normative collective action efficacy is expected to drive the sedative effect of politicized identity on normative collective action intentions, with politicized identity being positively associated with non‐normative collective action efficacy, which in turn should be negatively associated with normative collective action intentions.

As for non‐normative collective action, with regard to non‐politicized identity (Study2), we also expect positive indirect associations via increased perceived efficacy perceptions, except for non‐normative collective action efficacy. This time, non‐normative collective action efficacy is expected to mediate the mobilizing effect of non‐politicized identity on normative collective action intentions, with non‐politicized identity being negatively associated with non‐normative collective action efficacy, which in turn should be negatively associated with normative collective action intentions.

With respect to non‐normative collective action, we predict that politicized identity (Studies 1 and 2) will show positive indirect associations via increased group efficacy and non‐normative collective action efficacy. Conversely, we anticipate negative indirect associations through increased normative collective action efficacy and internal locus of control (which should be both positively predicted by politicized identity, and in turn be negatively associated with non‐normative collective action intentions).

Regarding non‐normative collective action and non‐politicized identity (Study 2), we expect the same negative indirect associations via increased normative collective action efficacy and internal locus of control. Additionally, we hypothesize a negative indirect association via decreased non‐normative collective action efficacy, with the latter being positively associated with non‐normative collective action intentions. Finally, we expect a positive indirect association via group efficacy, such that non‐politicized identity is positively associated with group efficacy, which, in turn, is positively associated with non‐normative collective action.

**FIGURE 1:**
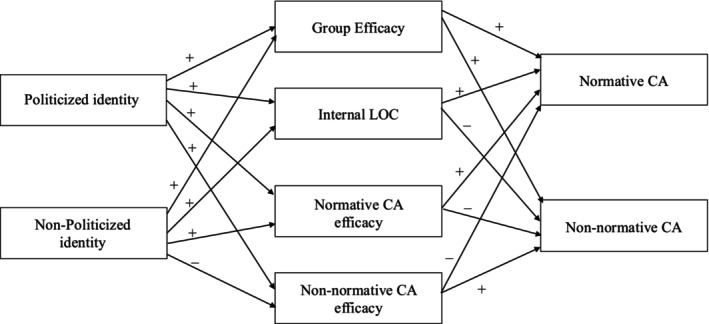
Hypothesized relationships among the variables. CA, collective action; LOC, locus of control.

Hypothesized relationships among the variables of Studies 1 and 2 are portrayed in Figure 1.

## STUDY 1

We conducted our first study in the Chilean context with a sample of Chilean students. Given that politicized identities are stronger predictors of collective action compared to non‐politicized identities (van Zomeren et al., 2008), we investigated a politicized identity relevant to this context. We focused on social identification with political organizations and parties that support students' rights in Chile, an identity inherently linked to political agendas. Between 2011 and 2013, the period during which these data were collected (2012), Chile experienced significant student protests triggered by an increase in government funding for non‐traditional education, eliciting widespread discontent among students demanding access to free and quality public education. Thousands of high school and university students marched in the capital Santiago and other cities, often resulting in violent clashes with police. This context provided an ideal context to investigate the role of efficacy perceptions in explaining the transition from who we are to what we are willing to do for social change. The investigation was conducted within a SIMCA framework (van Zomeren et al., 2008), with group‐based anger, representing the most proximal exemplification of affective injustice (key mediator alongside efficacy identified by SIMCA), serving as a control variable in the relationship between the mediators and the outcome variables. Including group‐based anger as a control variable is crucial for discerning the unique contributions of distinct forms of perceived efficacy in predicting collective action.

### Method

Data, codebook, and R script of this study are openly available at: https://osf.io/j2t7k/?view_only=bf1510b6720c4d4aa79bed8162eb5450.

#### Participants and procedure

We relied on a convenience sample of university students enrolled in a Chilean public University. Participants were recruited by the last author of this article, who administered them an online questionnaire. Participation was on a voluntary basis, without any compensation in exchange. In the informed consent of the study, participants were explicitly informed that the questionnaire was meant to investigate the relationship between identification with political organizations or parties that advocate for student rights in Chile and collective action intentions; they were also informed of the anonymity of their responses and the possibility to withdraw at any time. Participants who did not provide consent or had more than 30% of missing data were excluded from the analyses (*N* = 18). A total of 587 university students (men = 305; women = 221; all identifying between 18 and 26 years old) accepted to participate in the study and were therefore included in the analyses. We employed the *N:q* rule, which is the ratio between the study's cases and the number of model parameters that require statistical estimates (Kline, 2011). Our study's model includes one predictor, four parallel mediators, two dependent variables, and one control variable; the total number of estimated parameters is 29, suggesting that a sample of 587 achieves a parameters ratio of 20:1, that is considered optimal.

#### Measures

Unless otherwise indicated, all measures were answered on 7‐point scales (1 = Totally disagree, 7 = Totally agree). Full measures are presented in the Supporting Information.

##### Predictor variable: social identification

###### Politicized identity

We assessed the degree to which participants identified with political organizations or parties that support students' rights in Chile with four items, adapted from widely employed measures assessing politicized identification (van Zomeren et al., 2012). Respondents rated the items (e.g., “I feel strong ties to political organisations/parties that support student rights in Chile”) on a 7‐point scale, going from 1 = Totally disagree to 7 = Totally agree (*α* = .88).

##### Mediator variables: efficacy perceptions

###### Group efficacy

Four items, adapted from van Zomeren et al. (2004), were used to assess perceived group efficacy. Participants rated their perceived ability, as students of public universities in Chile, to promote change in various aspects related to their education and student life (e.g., “We, the students of Chile's public universities, can change the conditions of students in Chile for the better”; *α* = .90).

###### Collective action efficacy

Collective action efficacy was measured by eight items and consistently with more recent operationalizations of the construct (Saab et al., 2016). Participants expressed their level of agreement on the effectiveness of normative (e.g., “A legal demonstration would help change the government's position on this issue”; *α* = .90) and non‐normative (“Only a violent demonstration can change the government's mind”; *α* = .92) collective action.

###### Internal locus of control

It was measured with four items (e.g., “My life is determined by my own actions”), consistent with more recent measurements of the construct (Botha & Dahmann, 2023), which participants rated on a scale going from 1 = Totally disagree to 8 = Totally agree (*α* = .76).

##### Outcome variables: collective action

###### Normative and non‐normative collective action intentions

Participants' willingness to engage in collective action aimed at improving the current situation for students in public universities in Chile was assessed via seven items (adapted from van Zomeren et al., 2011): five items measured normative collective action (e.g., “I would vote for a candidate who is willing to improve the current situation for students in public universities in Chile”; *α* = .83), two items measured non‐normative collective action (e.g., “I would be willing to engage in violent protest if it were to improve conditions for students at public universities in Chile”; *ρ* = .87).

##### Covariate

###### Group‐based anger

We assessed anger with a single item (adapted from van Zomeren et al., 2004), by asking participants to rate how much anger they felt when thinking that Chilean students deserve a better education.

### Results

All analyses were performed in R (R Core Team, 2024). Descriptives and correlations among the variables are presented in Table 1–Panel A.

**TABLE 1:** Means, standard deviations, and correlations among the variables of Study 1 (Panel A) and Study 2 (Panel B).

		*M* (*SD*)	Politicized identity	Correlations						
				Internal LOC	Group efficacy	Normative CA efficacy	Non‐normative CA efficacy	Group‐based anger	Normative CA intentions	Non‐norm CA intentions
Panel A	Politicized identity	3.45 (2.18)	–							
	Internal LOC	5.92 (1.40)	0.04	–						
	Group efficacy	5.64 (1.46)	0.24***	.05	–					
	Normative CA efficacy	3.60 (1.90)	0.15***	0.09*	.29***	–				
	Non‐normative CA efficacy	2.91 (1.93)	0.29***	−.11**	.31***	.03	–			
	Group‐based anger	2.17 (1.74)	0.01	−.06	−.06	−.05	.05	–		
	Normative CA intentions	5.89 (1.35)	0.32***	−.03	.55***	.25***	.24***	−.16***	–	
	Non‐normative CA intentions	3.84 (2.39)	0.35***	−.04	.34***	−.02	.74***	−.01	.36***	

		*M* (*SD*)	Politicized identity	Correlations							
				Non‐politicized id	Internal LOC	Group efficacy	Normative CA Efficacy	Non‐normative CA Efficacy	Group‐Based Anger	Normative CA Intentions	Non‐norm CA Intentions
Panel B	Politicized identity	3.85 (1.55)	–								
	Non‐politicized id	4.64 (1.52)	0.03	–							
	Internal LOC	4.69 (1.15)	0.05	.13**	–						
	Group efficacy	5.52 (1.26)	0.18***	0.11*	.19***	–					
	Normative CA efficacy	3.82 (1.65)	0.11*	.17***	.02	.26***	–				
	Non‐normative CA efficacy	2.26 (1.55)	0.20***	−.13**	.00	.04	−.03	–			
	Group‐based anger	4.86 (1.83)	0.13**	.08	0.10*	.09	.08	.22***	–		
	Normative CA intentions	5.51 (1.23)	0.22***	.01	.05	.21***	.25***	.03	.17***	–	
	Non‐normative CA intentions	1.87 (1.48)	0.18***	−.19***	−0.10*	.01	−.03	.54***	.07	.03	–

Abbreviations: CA, collective action; LOC, locus of control.

*** *p* < .001.

** *p* < .01.

* *p* < .05.

To test H1, first of all, we ran regression models to test the direct effects of politicized identity on normative and non‐normative collective action, respectively. In line with our predictions, politicized identity had a significant and positive direct effect on both normative, *b* = .20, *SE* = .03, *p* < .001, and non‐normative, *b* = .38, *SE* = .04, *p* < .001, collective action intentions.

Following, to test H3 and H5, we ran a mediation model, employing the R package *lavaan* (Rosseel, 2012). In the model, politicized identity was the predictor, normative and non‐normative collective action were the dependent variables, while internal locus of control, group efficacy, and normative and non‐normative collective action efficacy were simultaneous mediators. Group‐based anger was used in the model as a control in the relationship between the mediators and the outcome variables. To identify mediating processes, we conducted a bias‐corrected bootstrapping procedure (Preacher & Hayes, 2008) with 10,000 resamples. To determine the statistical significance of unstandardized indirect effects, we used 95% confidence intervals; standardized effects were utilized to gauge the magnitude of the indirect effect.

**FIGURE 2:**
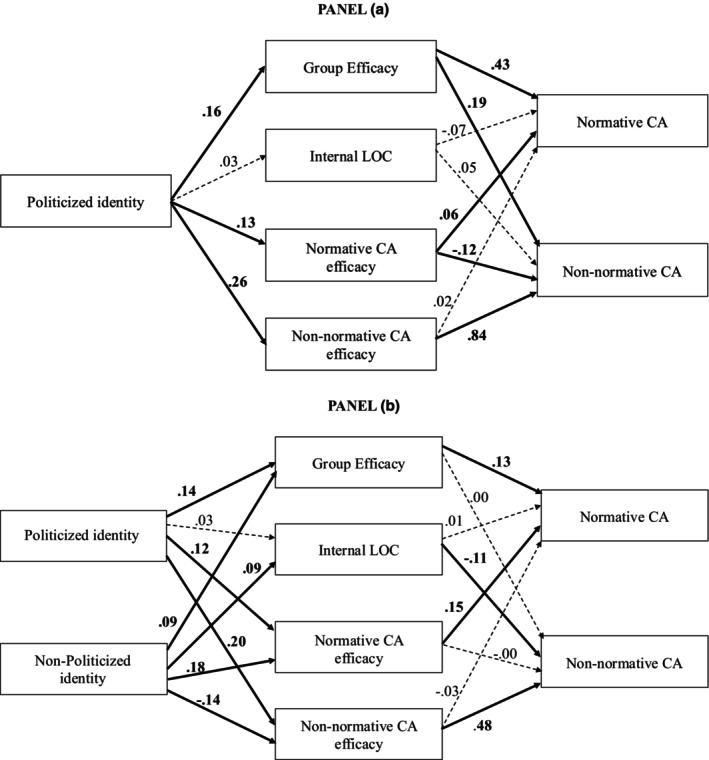
Direct relationship (unstandardized estimates) among the variables in the mediation model of Study 1 (Panel a) and Study 2 (Panel b). Effects are unstandardized. Statistically significant effects (in bold) determined by 95% bias‐corrected bootstrapped confidence interval (10,000 bootstrapped samples). CA, collective action; LOC, locus of control.

Relationships among the variables in the model are presented in Table 2–Panel A (see Figure 2–Panel a for a graphical representation). Results showed that the predictor, politicized identity, exhibited positive associations with group efficacy, as well as with normative and non‐normative collective action efficacy. Regarding the relationship between the mediators and the outcome variables, both group efficacy and normative collective action efficacy were positively associated with normative collective action intentions. Similarly, for non‐normative collective action intentions, both group and non‐normative collective action efficacy showed positive associations. Conversely, the association between normative collective action efficacy and non‐normative collective action intentions was reversed. Finally, the residual direct effect of politicized identity remained significant for both normative and non‐normative collective action intentions.

**TABLE 2:** Direct relationship (unstandardized estimates) among the variables in the mediation model of Study 1 (Panel A) and Study 2 (Panel B).

	Mediators				Outcome variables	
	Internal LOC	Group efficacy	Normative CA efficacy	Non‐normative CA efficacy	Normative CA	Non‐normative CA
Panel A						
Predictor						
Politicized identity	0.03 [−0.028, 0.079]	**0.16 [0.106, 0.214]**	**0.13 [0.055, 0.203]**	**0.26 [0.184, 0.324]**	**0.12 [0.078, 0.161]**	**0.15 [0.090, 218]**
Mediators						
Internal LOC					−0.07 [−0.141, 0.000]	0.05 [−0.051, 0.140]
Group efficacy					**0.43 [0.322, 0.522]**	**0.19 [0.095, 0.283]**
Normative CA efficacy					**0.06 [0.012, 0.108]**	**−0.12 [−0.198, −0.050]**
Non‐normative CA efficacy					0.02 [−0.031, 0.074]	**0.84 [0.765, 0.906]**
Control						
Group‐based anger					**−0.11 [−0.166, −0.050]**	−0.06 [−0.134, 0.007]
Panel B						
Predictor						
Politicized identity	0.03 [−0.033, 0.106]	**0.14 [0.062, 0.212]**	**0.12 [0.018, 0.217]**	**0.20 [0.103, 0.301]**	**0.13 [0.043, 0.210]**	0.08 [−0.000, 0.157]
Non‐politicized identity	**0.09 [0.017, 0.171]**	**0.09 [0.009, 0.167]**	**0.18 [0.073, 0.284]**	**−0.14 [−0.240, −0.039]**	−0.05 [−0.123, 0.030]	**−0.11 [−0.199, −0.019]**
Mediators						
Internal LOC					0.01 [−0.096, 0.125]	**−0.11 [−0.223, −0.005]**
Group efficacy					**0.13 [0.028, 0.224]**	0.00 [−0.093, 0.100]
Normative CA efficacy					**0.15 [0.075, 0.220]**	−0.00 [−0.080, 0.079]
Non‐normative CA efficacy					−0.03 [−0.116, 0.052]	**0.48 [0.363, 0.599]**
Control						
Group‐based anger					**0.09 [0.026, 0.151]**	−0.03 [−0.085, 0.036]

*Note*: Effects are unstandardized. Statistically significant effects (in bold) determined by 95% bias‐corrected bootstrapped confidence interval (10,000 bootstrapped samples).

Abbreviations: CA, collective action; LOC, locus of control.

As for indirect effects, generally consistent with H3 and H5, results revealed several indirect paths linking politicized identity to the outcome variables (see Table 3–Panel A for indirect effects). Specifically, as for H3, politicized identity was indirectly associated with greater normative collective action intentions, via increased group and normative collective action efficacy. Consistently, with respect to H5, we found similar paths for non‐normative collective action, with politicized identity being indirectly associated with greater non‐normative collective action intentions via increased group and non‐normative collective action efficacy. Finally, we also found a negative indirect effect of politicized identity on non‐normative collective action intentions via increased perception of normative collective action efficacy.^1^

**TABLE 3:** Indirect effects of the mediation model of Study 1 (Panel A) and Study 2 (Panel B).

	Predictors	Mediator	Outcome	Indirect effects	
				Unstandardized [95% CI]	Standardized
Panel A	Politicized identity	Group efficacy	Normative CA intentions	0.07 [0.040, 0.100]	.11
	Politicized identity	Normative CA efficacy	Normative CA intentions	0.01 [0.001, 0.016]	.01
	Politicized identity	Group efficacy	Non‐normative CA intentions	0.03 [0.014, 0.050]	.03
	Politicized identity	normative ca efficacy	Non‐normative CA intentions	−0.02 [−0.031, −0.005]	−.02
	Politicized identity	Non‐normative CA efficacy	Non‐normative CA intentions	0.21 [0.152, 0.275]	.20
Panel B	Politicized identity	Group efficacy	Normative CA intentions	0.02 [0.003, 0.038]	.02
	Politicized identity	Normative CA efficacy	Normative CA intentions	0.02 [0.002, 0.038]	.02
	Non‐politicized identity	Group efficacy	Normative CA intentions	0.01 [0.000, 0.028]	.01
	Non‐politicized identity	Normative CA efficacy	Normative CA intentions	0.03 [0.009, 0.049]	.03
	Politicized identity	Non‐normative CA efficacy	Non‐normative CA intentions	0.10 [0.048, 0.152]	.10
	Non‐politicized identity	Non‐normative CA efficacy	Non‐normative CA intentions	−0.07 [−0.118, −0.019]	−.07

*Note*: Only statistically significant effects (determined by 95% bias‐corrected bootstrapped confidence interval with 10,000 bootstrapped samples) are reported.

Abbreviation: CA, collective action.

### Discussion

The results of Study 1 provide support for the role of politicized identities in motivating individuals, both directly and indirectly through perceived efficacy, to undertake collective action (van Zomeren, 2008, 2012, 2018), whether normative or non‐normative. These findings extend the literature by broadly supporting our hypotheses regarding the unique contribution of distinct efficacy perceptions in driving the effects of politicized identity on collective action. They also shed light on the differing roles that efficacy perceptions may play in relation to normative and non‐normative collective action intentions. In this context, group and collective action efficacy emerged as key mediators capable of motivating individuals to undertake both conventional and more radical forms of collective action. Consistent with the hypotheses, these findings corroborate the idea that individuals are more likely to engage in collective actions when they perceive their group as capable of effecting change, regardless of whether the actions are normative or non‐normative. Moreover, as hypothesized, the efficacy of the specific action to be implemented—normative and non‐normative—plays a meaningful and distinct role in promoting or inhibiting intentions to carry it out. Firstly, normative collective action efficacy was positively associated with normative collective action intentions, and non‐normative collective action efficacy with non‐normative collective action intentions. Secondly, normative collective action efficacy seems to contribute in an additional way, exerting sedative effects on intentions towards non‐normative collective action, whereas non‐normative collective action efficacy did not significantly influence intentions towards normative actions. This result aligns with the idea that when individuals perceive normative actions as effective, they are more likely to engage in such actions and less likely to resort to non‐normative alternatives perceived as less appealing (Jiménez‐Moya et al., 2015; Wright, 2009). With respect to internal locus of control, contrary to expectations, it did not exhibit significant associations. This finding suggests that internal locus of control may not play a substantial role in mediating the relationship between politicized identity and collective action. However, it might also be that its effect is overshadowed by the influence of other determinants, such as group efficacy and collective action efficacy, which emerged as stronger predictors in this context.

Overall, these findings underscore the importance of group and collective action efficacy perceptions in the context of social identification and collective action, showing that while politicized identity represents a strong motivator of collective action, the type of efficacy perceived—whether normative or non‐normative—may significantly influence the form that this action takes.

## STUDY 2

To replicate and extend our findings, we conducted a second study in Italy, aimed at determining whether distinct social identities operate through similar or different efficacy perceptions in promoting normative and non‐normative collective action. We replicated the model from Study 1 and introduced identification with a non‐politicized identity as an additional predictor alongside politicized identification. Specifically, we included a measure assessing national identification, which represents a pre‐established form of social identity, an identity rooted in a sense of belonging derived from shared culture and history, rather than political agendas, and can therefore be independent of political movements (van Zomeren et al., 2008). Similar to Study 1, the context of reference (data were collected in 2012) consisted of widespread dissatisfaction, in this case, stemming from a massive financial crisis triggered by the bursting of a housing bubble, which led to a severe financial crisis in the US economy. This crisis gradually took on a global dimension due to financial contagion mechanisms and continued to involve several European countries due to the European sovereign debt crisis. Moreover, in the Italian context, the precarious economic situation was also exacerbated by political challenges, with Silvio Berlusconi, the Italian prime minister at the time, who in 2012 resigned from his position after losing parliamentary majority. Mario Monti, an economist and former European Commissioner, was appointed as the new Prime Minister, forming a technocratic government aimed at addressing Italy's economic issues through austerity measures and economic reforms to restore financial stability.

In such a context of nations impacted by the financial crisis, when national identity is meaningful to individuals, collective action efforts can serve as a powerful means to address the disadvantaged position of their nation, promoting mobilization (Stathi et al., 2019). Therefore, non‐politicized identities, such as national identity, alongside politicized identities, may represent a further key predictor of social change intentions via collective action. However, as anticipated, for non‐politicized identity, we expect that its effect will manifest in a manner that can protect the ingroup from further damage resulting from engaging in more radical actions.

### Method

Data, codebook, and R script of this study are openly available at: https://osf.io/j2t7k/?view_only=bf1510b6720c4d4aa79bed8162eb5450.

#### Participants and procedure

We relied on a convenience sample of Italian participants; three research assistants were in charge of data collection. Participants completed a paper and pencil questionnaire: they were informed about the anonymity of their responses, the possibility to withdraw at any time, and the study's purposes (i.e., investigating the relationship between their degree of non‐politicized (national) and politicized identification, respectively, and collective action intentions). All participants provided informed consent and completed more than 30% of the questionnaire; therefore, no one was excluded from the analyses, resulting in a total of 438 participants (men = 213, women = 224, other = 1; *M*_age_ = 38.29, *SD*_age_ = 16.25) included in the analyses. As for Study 1, we employed the *N:q* rule. This time our model includes two predictors, four parallel mediators, two dependent variables, and one control variable, for a total number of 35 estimated parameters, suggesting that a sample of 438 achieves a parameters ratio of 12:1, which is above the acceptable ratio of 10:1.

#### Measures

Unless otherwise indicated, all measures were answered on 7‐point scales (1 = Totally disagree, 7 = Totally agree). Full measures are presented in the Supporting Information.

##### Predictor variables: social identification

###### Politicized identity

We measured politicized identity with the same items as in Study 1, with the only difference being that participants rated their degree of identification with political organizations or parties that oppose government‐imposed economic reforms and measures (e.g., “I feel strong ties to political organizations/parties that support that oppose the austerity measures implemented by the Italian Government”; *α* = .85).

###### Non‐politicized identity

To measure the identification with non‐politicized identity, we employed four items (adapted from Stathi et al., 2019; van Zomeren et al., 2012) assessing national identification (e.g., “What my Italian identity represents is important to me”; *α* = .86).

##### Mediator variables: efficacy perceptions

###### Group efficacy

Group efficacy was measured by adapting the four items employed in Study 1 to the context of Study 2 (e.g., “We, Italians, can change the conditions of Italy for the better”; *α* = .88).

###### Collective action efficacy

Normative collective action efficacy was assessed via three out of the four items employed in Study 1 (e.g., “A legally authorized demonstration would help change the government's mind”; *α* = .82), while non‐normative collective action efficacy was measured using the first three items of Study 1; the fourth item was changed to better fit the Italian cultural context (i.e., “I would support illegal actions if they ultimately served to improve the conditions of Italians”; *α* = .87).

###### Internal locus of control

Internal locus of control was assessed with the same items of Study 1 (*α* = .76).

##### Outcome variables

###### Normative and non‐normative collective action intentions and support

We assessed participants' willingness to engage in collective actions aimed at improving the economic situation of Italian people with four items (same as Study 1, except for the fifth item which was not included) assessing normative collective action intentions (*α* = .68) and 2 items assessing non‐normative collective action intentions (same as Study 1; *ρ* = .83).

##### Covariate

###### Group‐based anger

We assessed group‐based anger with the same item employed in Study 1; only this time, participants were asked to rate how much anger they felt when thinking that Italians deserve a better life.

### Results

As for Study 1, analyses were performed in R (R Core Team, 2024). In Table 1–Panel B, we present descriptives and correlations among the variables assessed in Study 2.

Consistent with Study 1, we ran two regression models to test whether politicized (H1) and non‐politicized (H2) identity were associated with normative and non‐normative collective action, respectively. In line with H1, politicized identity had a positive direct effect on both normative, *b* = .17, *SE* = .04, *p* < .001, and non‐normative, *b* = .16, *SE* = .04, *p* < .001, collective action intentions. As for H2, it was partially confirmed: while non‐politicized identity had a negative direct effect on non‐normative collective action intentions, *b* = −.20, *SE* = .04, *p* < .001, the effect of non‐politicized identity on normative collective action intentions was nonsignificant, *b* = .01, *SE* = .04, *p* = .70.

To replicate the model tested in Study 1 (H3, H5), while also taking into account the role of non‐politicized identification (H4, H6), we ran another mediation model (R package *lavaan*; Rosseel, 2012). The model was the same as Study 1, only this time politicized and non‐politicized identities were simultaneous predictors. Again, the statistical significance of indirect effects was determined by 95% bias‐corrected bootstrapped confidence intervals (10,000 resamples) and we used standardized estimates to evaluate their magnitude.

Overall, results were consistent with Study 1 (Tables 2 and 3–Panel B). Politicized identity was positively related to group efficacy, normative, and non‐normative collective action efficacy. Similarly, non‐politicized identity was also positively associated with group efficacy and efficacy of normative collective action; moreover, it was positively related to internal locus of control and negatively related to non‐normative collective action efficacy. As for the relationship between the mediators and normative collective action intentions, we found that group efficacy and normative collective action efficacy were positively associated with normative collective action intentions. In the case of non‐normative collective action intentions, internal locus of control and non‐normative collective action efficacy had, respectively, a negative and a positive association with it. Finally, as for residual direct effects, politicized identity had a positive relationship with normative collective action intentions; non‐politicized identity had a negative relationship with non‐normative collective action (see Figure 2–Panel b for a graphical representation).

Indirect effects were again generally consistent with our hypotheses. We found positive indirect effects of politicized identity on normative collective action intentions, via increased group and normative collective action efficacy, respectively (H3); similar patterns emerged also for non‐politicized identity (H4). As for non‐normative collective action intentions, we found a positive indirect effect of politicized identity via increased perceptions of non‐normative collective action efficacy (H5). Finally, consistent with H6, non‐politicized identity was associated with lower non‐normative collective action intentions via decreased non‐normative collective action efficacy.^2^

### Discussion

Results demonstrated the unique contribution of different types of efficacy perceptions in explaining the association between social identification and collective action. Group and collective action efficacy confirm their prominent role in driving the mobilizing effects of social identification, in this case with both politicized and non‐politicized identities, on normative collective action intentions. However, concerning non‐normative collective action, only perceived collective action efficacy consistently proves effective, highlighting the significance of considering not only the agent of collective action but also the perception of the action's potential success. These findings extend Study 1, confirming the hypothesized differential association of politicized and non‐politicized identities with non‐normative collective action intentions, with an indirect positive association for politicized identity and an indirect negative association for non‐politicized identity. In other words, while politicized identity promotes the intention to engage in more radical collective actions through the perception of efficacy associated with the action itself, non‐politicized identity, through the same process, inhibits such radical intentions, exerting a sedative effect on non‐normative action intentions.

## GENERAL DISCUSSION

Extensive research has demonstrated that perceived efficacy serves as a mechanism for translating social discontent into activism (Marsh, 1977; Muller, 1972), employing various indices and often incorporating mixed contents (Bandura, 1982; Hamann et al., 2024). However, discerning the contribution of different forms of efficacy perceptions to the dynamics between identity and collective action remains challenging. The current research, through two cross‐sectional studies employing data collected in non‐WEIRD (Chile) and WEIRD contexts (Italy), aimed at advancing our comprehension of collective action processes by examining the association between both politicized (Studies 1 and 2) and non‐politicized identities (Study 2) with normative and non‐normative collective action intentions. In doing so, we tested the comparative mediating role of distinct forms of efficacy perceptions (group efficacy, normative and non‐normative action efficacy, internal locus of control).

Results consistently demonstrate the significant and unique contribution of efficacy perceptions in explaining the transition from who we are to what we are willing to do for social change, underscoring the importance of considering not only the agent of action (group efficacy) but also the perceived efficacy associated with the action itself (collective action efficacy). Moreover, they suggest the potential instrumental use of social identity, indicating that it may play a crucial role in promoting or inhibiting more radical forms of collective action.

This research significantly contributes to the current literature in several ways by providing insights into (a) how identification with distinct social identities relates to different efficacy perceptions and, in turn, how these efficacy perceptions relate to normative and non‐normative forms of collective action; (b) the unique contribution of distinct efficacy perceptions in driving the effects of social identification on collective action intentions; (c) differences between politicized and non‐politicized identities in associating with normative and non‐normative collective action intentions; (d) whether social identification with politicized and non‐politicized identities works through the same or different efficacy processes in predicting collective action. Moreover, the inclusion of internal locus of control, an efficacy construct extensively explored in the context of pro‐environmental actions, within a SIMCA framework (van Zomeren et al., 2008), represents a first step towards reconciling different bodies of literature by providing a comprehensive understanding of the mechanisms driving human behaviour across various domains.

Delving into the instrumental use of social identity, the goal orientation of identities, whether politicized or non‐politicized, is confirmed to be a relevant factor (van Zomeren et al., 2008), potentially influencing not only collective action intentions but also the type of action undertaken. The perception of an action as normative or non‐normative is not an objective consideration but is closely tied to the ingroup in question and its normative framework (Uysal et al., 2024), which dictates what is considered normative and what is not for the group. Similarly, the focus of identity, whether on the group or on its goals, should be reflected in the normative content of identity, guiding members with respect to what should be done and what should be avoided to conform to the group's prototype and its prescriptions (Turner et al., 1987).

Politicized identities may be driven to do whatever it takes to achieve their goals (Simon et al., 1998) as they are defined by the struggle for specific aims and may be willing to resort to radical means to achieve them. In contrast, non‐politicized identities, which are pre‐existing and based on group identification rather than goals (van Zomeren et al., 2008), tend to adhere to norms that primarily aim to protect the group itself. Non‐politicized identified members could strategically explore all socially acceptable means to improve their position before delving into radical actions that might further compromise the group (Jiménez‐Moya et al., 2015; Stathi et al., 2019), opting for them only when they perceive a lack of efficacy through normative strategies (Tausch et al., 2011). Thus, non‐politicized identities may be more conservative in their actions, preferring strategies that do not entail high risks.

This argumentation aligns with the “nothing to lose” strategy (Becker & Tausch, 2015; Scheepers et al., 2006), which posits that it is a low (vs. high) perception of efficacy that promotes participation in radical actions. However, while we agree that individuals may resort to extreme strategies when they have nothing left to lose, we argue that this is functionally linked to the type of identity orientation at play (and therefore its normative content) and, in parallel, the availability of alternative socially acceptable strategies. This is in line with the notion of social identity as a motivational driving force for the acquisition and maintenance of a positive social identity (Tajfel & Turner, 1979), which, in their case, is represented by the achievement of goals. On the other hand, non‐politicized identities, whose normative content primarily prescribes the protection of the group, are motivated to improve their position. However, before resorting to radical actions that could harm the group, they explore all feasible normative routes. Only when these strategies are perceived as ineffective might the “nothing to lose” perception come into play.

These findings also have important implications regarding the role of collective action efficacy. They suggest that in situations of dissatisfaction and crisis, the perception of the effectiveness of action can play a crucial role in promoting or mitigating more radical actions. In order to prevent such actions, it is essential to cultivate the belief in people that their norm‐compliant efforts can actually make a real impact. Those in positions of power can influence these perceptions by using a strategy of incentives and sanctions to direct behaviour towards desired outcomes, offering rewards for adhering to norms and discouraging extreme behaviour. On the one hand, such power can be instrumental in encouraging people towards normative actions that many might adhere to, as they entail lower associated risks, thus favouring the achievement of goals. On the other hand, the “carrot and stick” mechanism could be employed to maintain the status quo. It is crucial to carefully examine how power is exercised in these situations and what consequences, both positive and negative, it may have on social dynamics and change.

For a more comprehensive understanding of these dynamics, especially regarding the role of identity orientation and its normative content, future studies could explore the relationship between distinct identities, collective action efficacy, and both normative and non‐normative collective action intentions. This investigation should delve into how the normative content of identity moderates the relationship between social identities and collective action efficacy, influencing the perception of whether non‐normative actions are perceived as effective or not. Adding to this, conducting further studies to compare distinct forms of efficacy perceptions could be pivotal in fully grasping their nuanced impacts on individuals' intentions and behaviours concerning social change. For instance, there are additional forms worthy of consideration. van Zomeren et al. (2013) introduced the concept of participative efficacy beliefs, which reflects the perception that one's actions will or will not make a difference in achieving group goals through collective efforts. They posited participative efficacy beliefs as a unique predictor of collective action, distinct from perceptions of group and individual efficacy. Finally, future research might also consider the role of cultural context by systematically comparing WEIRD and non‐WEIRD societies, incorporating multiple samples to enable meaningful comparisons across these heterogeneous contexts. Such efforts would not only strengthen the external validity of the observed associations but also contribute to a more inclusive and globally relevant understanding of the dynamics underlying collective action.

We acknowledge some limitations. Our studies are correlational in nature and therefore do not allow us to infer causality, necessitating future studies that can experimentally and longitudinally investigate our assumptions. However, it is also important to recognize the potential bidirectionality of such relationships (Thomas et al., 2012), given the dynamic nature of collective action processes. Moreover, although SIMCA serves as a foundational model against which researchers develop their lines of research, it is not the only one available. The relationships analysed in this study could benefit from incorporating theoretical foundations derived from other models. For instance, the dynamic model of engagement in normative and non‐normative collective action proposed by Becker and Tausch (2015) offers valuable insights into the role of emotions. This model demonstrates that non‐normative collective action is more likely to emerge when protesters feel contempt—an emotion with distinct social features and functions—rather than anger.

In conclusion, this study offers valuable insights into the intricate relationship between social identification, efficacy perceptions, and collective action, highlighting the different pathways through which individuals engage in social change. By addressing gaps between different theoretical frameworks and shedding light on the nuanced dynamics at play, it paves the way for future research aimed at further unraveling the complexities of individuals' behaviour in the pursuit of social change.

## AUTHOR CONTRIBUTIONS

**Alice Lucarini:** Conceptualization; methodology; data curation; formal analysis; visualization; writing – original draft. **Veronica Margherita Cocco:** Conceptualization; methodology; writing – original draft. **Loris Vezzali:** Conceptualization; writing – original draft; supervision. **Terri Mannarini:** Investigation; writing – review and editing. **Huseyin Çakal:** Data curation; investigation; writing – review and editing; supervision.

## FUNDING INFORMATION

This research was partially funded by an UKRI grant "A Participatory Psychosocial Care Approach to Mental Health Colombia Grant Number: ES/V013394/1 awarded to the corresponding author Huseyin Çakal.

## CONFLICT OF INTEREST STATEMENT

The authors declare no conflict of interest.

## INFORMED CONSENT

The authors declare that informed consent was obtained.

## Supporting information


Data S1.

## Data Availability

The data that support the findings of this study are openly available at: https://osf.io/j2t7k/?view_only=bf1510b6720c4d4aa79bed8162eb5450.

## Footnotes

^1^
In Study 1, even when not accounting for group‐based anger as a control variable, all paths were replicated.

^2^
In Study 2, even when not accounting for anger as a control variable, all paths were replicated, and we found an additional negative indirect effect linking non‐politicized identity to non‐normative collective action via increased internal locus of control; while the residual direct effect of politicized identity on non‐normative collective action remained significant.
