# Supplementary material for: From who we are to what we are willing to do for social change: The action‐bound role of efficacy perceptions
Source: Br J Soc Psychol. 2025 Jun 16;64(3):e12910. doi: 10.1111/bjso.12910 (PMC12169075; doi:10.1111/bjso.12910)
Supplement: Supplementary file 1 — Data S1. [file BJSO-64-0-s001.docx]

**Supplementary Materials**

**From Who We Are to What We Are Willing to Do for Social Change:**

**The Comparative Action-Bound Role of Perceived Efficacy Perceptions**

**in Normative and Non-Normative Collective Action**

**Items employed in Study 1**

**Politicized identity**

- Even though I am not, I see myself as a member of the political organisation that supports the rights of students in Chile.
- I feel strong ties to political organisations/parties that support student rights in Chile.
- I identify with members of a political organisation/party that supports student rights in Chile.
- I am very proud of the political organisations/parties that support students' rights in Chile.

**Group efficacy**

- We, the students of public universities in Chile, can change the current laws regarding fees by our own efforts.
- We, the students of Chile's public universities, can change the conditions of students in Chile for the better.
- Working together with other students, we can change the conditions for students in public universities in Chile.
- If you participate in an effort to change the conditions of students in Chile's public universities for the better, you can make a difference.

**Internal locus of control**

- When I make plans, I am pretty sure I can make them work.
- Normally I am able to protect my personal interests.
- I can determine almost everything that will happen in my life.
- My life is determined by my own actions.

**Efficacy of normative collective action**

- A legal demonstration would help change the government's position on this issue.
- A petition would contribute to changing the government's position on this issue.
- A public meeting would help change the government's position on this issue.
- A legal march would help change the government's position on this issue.

**Efficacy of non-normative collective action**

- Only a violent demonstration can change the government's mind.
- Violence will be an effective way to change the government's mind.
- We must use force and violence to change the government's mind on the issue.
- If it would help, we should confront the police or security forces.

**Group-based anger**

- When it is claimed that the students of Chile's public universities deserve to have an education to what extent do you feel: Anger.

**Normative collective action intentions**

- I would vote for a candidate who is willing to improve the current situation for students in public universities in Chile.
- I would be willing to sign a petition to improve the current situation for students in public universities in Chile.
- I would be willing to participate in a project to improve the current situation for students in public universities in Chile.
- I would you be willing to participate in a peaceful protest to improve the current situation of students in Chile's public universities.
- I would be willing to participate in an organisation that defends the rights of students at public universities in Chile.

**Non-normative collective action intentions**

- I would be willing to face the police in a protest to improve conditions for students at public universities in Chile.
- I would be willing to engage in violent protest if it were to improve conditions for students at public universities in Chile.

**Items employed in Study 2**

**Politicized identity**

- Even though I am not, I see myself as a member of a political organization that opposes the austerity measures and economic reforms imposed by the Italian Government.
- I feel strong ties to political organisations/parties that support that oppose the austerity measures implemented by the Italian Government.
- I identify with members of a political organisation/party that fights against the economic reforms decided by the Government.
- I am very proud of the political organisations/parties that do not approve of the Government's economic decisions.

**Non-politicized identity**

- I am proud to think of myself as Italian.
- What my Italian identity represents is important to me.
- feel a connection with other Italians.
- I feel a certain commitment towards Italy.

**Group efficacy**

- We, Italians, can change the current state of things by our own efforts.
- We, Italians, can change the conditions of Italy for the better.
- Working together with other Italians, we can change the conditions for our Country and our people.
- If you participate in an effort to change the conditions of Italy for the better, you can make a difference.

**Internal locus of control**

- When I make plans, I am pretty sure I can make them work.
- Normally I am able to protect my personal interests.
- I can determine almost everything that will happen in my life.
- My life is determined by my own actions.

**Efficacy of normative collective action**

- A legal demonstration would help change the government's position on this issue.
- A petition would contribute to changing the government's position on this issue.
- A public meeting would help change the government's position on this issue.

**Efficacy of non-normative collective action**

- Only a violent demonstration can change the government's mind.
- Violence will be an effective way to change the government's mind.
- We must use force and violence to change the government's mind on the issue.
- I would support illegal actions if they ultimately served to improve the conditions of Italians.

**Group-based anger**

- When it is claimed that Italians deserve a better life, to what extent do you feel: Anger.

**Normative collective action intentions**

- I would vote for a candidate who is willing to improve the economic situation of Italians.
- I would be willing to sign a petition to improve the economic situation of Italians.
- I would be willing to participate in a project to improve the economic situation of Italians.
- I would you be willing to participate in a peaceful protest to improve the economic situation of Italians.

**Non-normative collective action intentions**

- I would be willing to face the police in a protest to improve the economic situation of Italians.
- I would be willing to engage in violent protest if it were to improve the economic situation of Italians.
